# TonB Energy Transduction Systems of *Riemerella anatipestifer* Are Required for Iron and Hemin Utilization

**DOI:** 10.1371/journal.pone.0127506

**Published:** 2015-05-27

**Authors:** HeBin Liao, XingJun Cheng, DeKang Zhu, MingShu Wang, RenYong Jia, Shun Chen, XiaoYue Chen, Francis Biville, MaFeng Liu, AnChun Cheng

**Affiliations:** 1 Avian Disease Research Center, College of Veterinary Medicine of Sichuan Agricultural University, Ya’an, Sichuan, People’s Republic of China; 2 Institute of Preventive Veterinary Medicine, Sichuan Agricultural University, Chengdu, Sichuan, People’s Republic of China; 3 Key Laboratory of Animal Disease and Human Health of Sichuan Province, Sichuan Agricultural University, Chengdu, Sichuan, People’s Republic of China; 4 Unité des Infections Bactériennes Invasives, Institut Pasteur, Paris, France

## Abstract

*Riemerella anatipestifer* (*R*. *anatipestifer*) is one of the most important pathogens in ducks. The bacteria causes acute or chronic septicemia characterized by fibrinous pericarditis and meningitis. The *R*. *anatipestifer* genome encodes multiple iron/hemin-uptake systems that facilitate adaptation to iron-limited host environments. These systems include several TonB-dependent transporters and three TonB proteins responsible for energy transduction. These three *tonB* genes are present in all the *R*. *anatipestifer* genomes sequenced so far. Two of these genes are contained within the *exbB-exbD-tonB1* and *exbB-exbD-exbD-tonB2* operons. The third, *tonB3*, forms a monocistronic transcription unit. The inability to recover derivatives deleted for this gene suggests its product is essential for *R*. *anatipestifer* growth. Here, we show that deletion of *tonB1* had no effect on hemin uptake of *R*. *anatipestifer*, though disruption of *tonB2* strongly decreases hemin uptake, and disruption of both *tonB1* and *tonB2* abolishes the transport of exogenously added hemin. The ability of *R*. *anatipestifer* to grow on iron-depleted medium is decreased by tonB2 but not *tonB1* disruption. When expressed in an *E*. *coli* model strain, the TonB1 complex, TonB2 complex, and TonB3 protein from *R*. *anatipestifer* cannot energize heterologous hemin transporters. Further, only the TonB1 complex can energize a *R*. *anatipestifer* hemin transporter when co-expressed in an *E*. *coli* model strain.

## Introduction

*R*. *anatipestifer*, belonging to the family *Flavobacteriaceae* and genus *Riemerella*, is a gram-negative bacterium. It is one of the most harmful pathogens to the duck, chicken, geese and turkey industries around the world, as it can quickly become endemic and is difficult to eradicate [1]. So far, at least 21 serotypes have been identified in different countries with no cross-protection between them. Among them, serotypes 1, 2, 3, 5, and 15 are the major pathogens affecting the duck industry [2–4]. Although *Reimerellosis* causes serious economic losses, the pathogenic mechanisms and the virulence factors of *R*. *anatipestifer* remain mostly unknown. At present, OmpA [5], VapD [6], CAMP cohemolysin [7], TbdR1 [8] and siderophore-interacting protein (Sip) [9] have been identified as potential virulence factors. Among these, it was shown that the *sip* deletion mutant had significantly impaired iron uptake and reduced toxicity in ducklings compared to the wild type [9]. This suggested that *R*. *anatipestifer* can utilize siderophores to capture iron from its surroundings. The TonB-dependent receptor TbdR1 has been shown to be involved in hemin and iron acquisition, and the LD_50_ value of the *tbdR1* mutant is approximately 45-fold higher than that of the wild-type strain [8]. This result suggests that the hemin/iron uptake process plays an important role in the pathogenesis of *R*. *anatipestifer*.

Iron is an essential element for most organisms. In bacteria, iron acts as a catalytic center for many biological enzymes involved in electron transport, peroxide reduction, and nucleotide biosynthesis [10–12]. However, iron availability is often limited in both the environment and the host. In the host, some iron sources are present in the form of iron-containing proteins such as lactoferrin, transferrin, and ferritin [11]. However, most iron sources are in the form of hemin, which is mainly incorporated into hemoproteins [13]. Scavenging iron and hemin prevents toxic effects on the host and plays an important role in nutritional immunity, which aids in the prevention of pathogen infection by limiting iron availability [14].

Pathogens have developed several strategies to withstand host nutritional immunity. For example, many vertebrate pathogens encode transport systems that enable the use of iron-loaded proteins [15] or hemin-loaded proteins as iron sources [16,17]. The known hemin uptake systems of gram-negative bacteria include direct hemin uptake systems and hemophore-mediated hemin uptake systems [18]. Briefly, in the classical model of hemin uptake in gram-negative bacteria, TonB-dependent outer membrane receptors bind and transport hemin through the outer membrane [19]. Inside the periplasm, hemin is bound by a specific protein that transports it to an inner membrane ABC transporter [20–22]. Inside the cell, hemin is degraded by hemin oxygenase or hemin degrading protein to release Fe^2+^ to support iron requirements [20–22].

In addition to hemin transport, TonB also energizes many other outer membrane transporters responsible for iron-loaded siderophores, vitamin B12, nickel complexes and carbohydrates in most gram-negative bacteria [23–26]. In some bacteria, two or more versions of the TonB complex have been identified [27–30]. In the case of *Serratia marcescens*, the specificity of a TonB homolog, HasB, for a hemophore-dependent heme uptake system was demonstrated [31]. However, the mechanism of redundancy of TonB proteins in bacteria is not fully understood. In addition to functioning in nutrient transport, it has also been shown that TonB is involved in the transduction of environmental signals, is essential for virulence, and functions in biofilm formation [32–35].

Sequence comparisons allowed the identification of genes predicted to encode three TonB-like proteins and 31 putative TonB-dependent receptors in the *R*. *anatipestifer* genome [8]. However, the functions of the TonB proteins in iron and hemin transportation have not been investigated. Functional identification of these TonB proteins may support further understanding of the iron and hemin transport systems and is also important for understanding the pathogenic mechanisms of *R*. *anatipestifer*. Investigation of these TonB proteins may additionally lead to new strategies for the prevention and treatment of infectious serositis in ducklings caused by *R*. *anatipestifer*. In this study, we investigated the roles of the three TonB proteins of *R*. *anatipestifer* strain ATCC11845 in hemin and iron utilization.

## Materials and Methods

### Bacterial Strains and Plasmids Used in This Study

The bacterial strains and plasmids used in this study are listed in Table 1.

**Table 1:** Strains and plasmids used in this study.

*E*. *coli* strains	Genotype	Source or reference
JP313	*araD139 relA rpsL 150 thi flb5301 (lacU 139) deo7 ptsF25 Δara 174*	Laboratory collection
JP313 pBAD24	JP313 carrying pBAD24, AmpR	This study
JP313 pBAD24::*tonB1*	JP313 carrying pBAD24::*tonB1*, AmpR	This study
JP313 pBAD24::*tonB2*	JP313 carrying pBAD24::*tonB2*, AmpR	This study
JP313 pBAD24::*tonB3*	JP313 carrying pBAD24::*tonB3*, AmpR	This study
C600 *ΔhemA*	C600 (F*- thr leu lacY thi supE ΔhemA*::Km), KmR	[54]
C600 *ΔhemA* pBAD24	C600*ΔhemA* carrying pBAD24, AmpR	This study
C600*ΔhemA tonB*::Tn*10*	C600*ΔhemA*::Km *tonB trp*::Tn*10*, KmR, TetR	[54]
C600*ΔhemA tonB*::Tn*10* pBAD24	C600*ΔhemA tonB*::Tn*10* carrying pBAD24, KmR, TetR, AmpR	This study
C600*ΔhemA tonB*::Tn*10* pBAD24::*tonB1*	C600*ΔhemA tonB*::Tn*10* carrying pBAD24:: *tonB1*, KmR, TetR, AmpR	This study
C600*ΔhemA tonB*::Tn*10* pBAD24::*tonB2*	C600*ΔhemA tonB*::Tn*10* carrying pBAD24:: *tonB2*, KmR, TetR, AmpR	This study
C600*ΔhemA tonB*::Tn*10* pBAD24::*tonB3*	C600*ΔhemA tonB*::Tn*10* carrying pBAD24:: *tonB3*, KmR, TetR, AmpR	This study
C600*ΔhemA tonB*::Tn*10* pBAD24::*exbB1-exbD1-tonB1*	C600*ΔhemA tonB*::Tn*10* carrying pBAD24:: *exbB1-exbD1-tonB1*, KmR, TetR, AmpR	This study
C600*ΔhemA tonB*::Tn*10* pBAD24::*exbB2-exbD21-exbD22-tonB2*	C600*ΔhemA tonB*::Tn*10* carrying pBAD24:: *exbB2-exbD21-exbD22-tonB2*, KmR, TetR, AmpR	This study
C600*ΔhemA tonB*::Tn*10* pAM238::*OMhemR*_*RA*_ pBAD24	C600*ΔhemA tonB*::Tn*10* pAM238::*OMhemR*_*RA*_ carrying pBAD24, KmR, TetR, AmpR, SpcR	This study
C600*ΔhemA tonB*::Tn*10* pAM238::*OMhemR*_*RA*_ pBAD24::*tonB1*	C600*ΔhemA tonB*::Tn*10* pAM238::*OMhemR*_*RA*_ carrying pBAD24:: *tonB1*, KmR, TetR, AmpR, SpcR	This study
C600*ΔhemA tonB*::Tn*10* pAM238::*OMhemR*_*RA*_ pBAD24::*tonB2*	C600*ΔhemA tonB*::Tn*10* pAM238::*OMhemR*_*RA*_ carrying pBAD24:: *tonB2*, KmR, TetR, AmpR, SpcR	This study
C600*ΔhemA tonB*::Tn*10* pAM238::*OMhemR*_*RA*_ pBAD24::*tonB3*	C600*ΔhemA tonB*::Tn*10* pAM238::*OMhemR*_*RA*_ carrying pBAD24:: *tonB3*, KmR, TetR, AmpR, SpcR	This study
C600*ΔhemA tonB*::Tn*10* pAM238::*OMhemR*_*RA*_ pBAD24::*exbB1-exbD1-tonB1*	C600*ΔhemA tonB*::Tn*10* pAM238::*OMhemR*_*RA*_ carrying pBAD24:: *exbB1-exbD1-tonB1*, KmR, TetR, AmpR, SpcR	This study
C600*ΔhemA tonB*::Tn*10* pAM238::*OMhemR*_*RA*_ pBAD24::*exbB2-exbD21-exbD22-tonB2*	C600*ΔhemA tonB*::Tn*10* pAM238::*OMhemR*_*RA*_ carrying pBAD24:: *exbB2-exbD21-exbD22-tonB2*, KmR, TetR, AmpR, SpcR	This study
XL1-BLUE	F^-^ *supE44 hdsR17 recA1 endA1 gyrA46 thi relA1* lac^-^ F’ *proAB* ^-^ *lacI* ^q^ *lacZΔM15* Tn*10*, TetR	Laboratory collection
S17-1	*Thi-1 thr leu tonA lac Y supE recA*::RP4-2-Tc::Mu KmR	[55]
S17-1 pEX18GM::*tonB1ued*	S17-1 carrying pEX18GM::*tonB1ued*, KmR,GenR	This study
S17-1 pEX18GM::*tonB2usd*	S17-1 carrying pEX18GM::*tonB2usd*, KmR,GenR	This study
***Riemerella anatipestifer* strains**	**Genotype**	**Source or reference**
*R*. *anatipestifer* ATCC11845	ATCC11845	Laboratory collection
*R*. *anatipestifer* CH-1	Seroytype 1	Laboratory collection
*R*. *anatipestifer* ATCC11845 *ΔtonB1*::*ermR*	*R*. *anatipestifer* ATCC11845 *tonB1*::*ermR*,ErmR	This study
*R*. *anatipestifer* ATCC11845 *ΔtonB2*::*spcR*	*R*. *anatipestifer* ATCC11845 *tonB2*::*spcR*,SpcR	This study
*R*. *anatipestifer* ATCC11845 *ΔtonB1*::*ermR ΔtonB2*::*spcR*	*R*. *anatipestifer* ATCC11845 *tonB1*::*ermR tonB2*::*spcR*, ErmR SpcR	This study
**Plasmids**	**Genotype**	**Source or reference**
pBAD24	pBR322 araC, arabinose-inducible promoter, AmpR	Laboratory collection
pBAD24::*tonB1*	pBAD24 carrying *tonB1* from *R*. *anatipestifer* ATCC11845, AmpR	This study
pBAD24::*tonB2*	pBAD24 carrying *tonB2* from *R*. *anatipestifer* ATCC11845, AmpR	This study
pBAD24::*tonB3*	pBAD24 carrying *tonB3* from *R*. *anatipestifer* ATCC11845, AmpR	This study
pBAD24::*tonB1 his*	pBAD24 carrying *tonB1* adding his tag, AmpR	This study
pBAD24::*tonB2 his*	pBAD24 carrying t*onB2* adding his tag, AmpR	This study
pBAD24::*tonB3 his*	pBAD24 carrying *tonB3* adding his tag, AmpR	This study
pBAD24::*exbB1-exbD1-tonB1*	pBAD24 carrying *exbB1-exbD1-tonB1* from *R*. *anatipestife*r ATCC11845, AmpR	This study
pBAD24::*exbB2-exbD21-exbD22-tonB2*	pBAD24 carrying *exbB2-exbD21-exbD22-tonB2* from *R*. *anatipestifer* ATCC11845, AmpR	This study
pAM238	pSC101 origin, SpcR	[52]
pAM238::*OMhemR*_*RA*_	pAM238 carrying *OMhemR*_*RA*_ from *R*. *anatipestife*r ATCC11845, SpcR	This study
pEX18GM	*oriT* ^*+*^, *sacB* ^*+*^, gene replacement vector with MCS from pUC18, GenR	[56]
pEX18GM::*tonB1ued*	pEX18GM carrying *tonB1ued* from *R*. *anatipestifer* ATCC11845 and *R*. *anatipestifer* CH-1, GenR	This study
pEX18GM::*tonB2usd*	pEX18GM carrying *tonB2usd* from *R*. *anatipestifer* ATCC11845 and pAM238, GenR	This study

AmpR, ampicillin resistance; GenR, gentamicin resistance; KmR, kanamycin resistance; TetR, tetracycline resistance; ErmR, erythromycin resistance; SpcR, spectinomycin resistance.

### Media and Growth Conditions

Hemin, *δ*-aminolevulinic acid (δ-ala) and 2,2’-dipyridyl (Dip) were obtained from Sigma Chemical (Sigma, China). Hemin was dissolved immediately before use in 0.1 N NaOH, Dip was dissolved in ethanol, and δ-ala was dissolved in distilled water. Hemin and δ-ala were filter-sterilized with 0.22-μm pore-size Millipore filters. *E*. *coli* strains were grown on LB medium (Sigma-Aldrich, Product Number: L3522) aerobically at 37°C. When required, δ-ala was used at a concentration of 50 μg/ml. Solid media contained 1.5% Difco agar. Iron-depleted medium for *E*. *coli* was obtained by the addition of Dip at a final concentration of 150 μM. Antibiotics for *E*. *coli* were added to the following final concentrations (μg/ml): Ampicillin (Amp), 100; Kanamycin (Km), 50; Spectinomycin (Spc), 50; Gentamicin (Gen), 20. Arabinose was added at 0.02% for induction of the pBAD promoter. IPTG was added at 0.5 mM for induction of pAM238 expression. *R*. *anatipestifer* was grown on LB plates supplemented with 5% defibrinated sheep blood [4] or TSA plates (Tryptone soy broth, TSB, containing 1.5% agar) [9] at 37°C under 5% CO_2_ atmosphere.

### *E*. *coli* Hemin-dependent Growth Assays and Iron-chelated Growth Assays

The tested *E*. *coli* strains, C600*ΔhemA tonB*::Tn*10* pAM238::*OMhemR*_*RA*_, harboring plasmid pBAD24 and pBAD24 derivatives containing *R*. *anatipestifer tonB* genes were grown for 5 h in LB medium containing δ-ala, Spc and Amp. Bacteria were collected, re-suspended in 1 ml Phosphate Buffered Saline (PBS) and centrifuged for 5 min at 5,000 g. This procedure was repeated three times to wash the bacteria. The OD values at 600 nm were measured for bacterial suspensions, which were then adjusted to 10,000 bacteria/ml (1 OD_600_ = 5×10^8^ bacteria). Then, 20-μl samples (approximately 200 bacteria) of the bacterial suspension were used to inoculate LB plates, LB plates containing 50 μg/ml δ-ala, and LB plates containing 20 μM hemin. Growth was recorded after 1 day incubation at 37°C. All experiments were performed in triplicate.

*E*. *coli* strains, C600*ΔhemA tonB*::Tn*10*, harboring a pBAD24 derivative containing an *R*. *anatipestifer tonB* gene, were inoculated in LB medium containing 50 μg/ml δ-ala and Amp and were grown for approximately 5 h. Then, bacteria were harvested and numerated as mentioned above. Approximately 200 bacteria were inoculated onto LB plates or iron-depleted plates which contained Dip at 150 μM. The growth was recorded after 1 day incubation at 37°C. All experiments were performed in triplicate.

### The Effect of *R*. *anatipestifer tonB* Knockouts on Hemin and Iron Uptake

*R*. *anatipestifer* ATCC11845 and the *tonB1*, *tonB2*, and *tonB1tonB2* mutant derivatives were inoculated onto LB plates containing 5% defibrinated sheep blood and incubated overnight at 37°C under 5% CO_2_ atmosphere. Bacteria were collected, re-suspended in 1 ml PBS and centrifuged for 5 min at 5,000 g. This operation was repeated three times to wash the bacteria. The OD_600_ values of the bacterial suspensions were measured and adjusted to 10,000 bacteria/ml (1 OD_600_ = 6×10^8^ bacteria). Then, 20 μl (approximately 200 bacteria) were inoculated onto LB plates, LB plates containing 20 μM hemin, LB plates containing 5% bovine serum or LB plates containing 5% bovine serum and 40 μM Dip. The growth was recorded after a 2-day-long incubation at 37°C under 5% CO_2_ atmosphere. All experiments were performed in triplicate.

### Genetic Techniques

*E*. *coli* cells were transformed by the calcium chloride method as described by Maniatis [36].

### DNA Manipulation

Small-scale plasmid DNA preparations were performed using a QIAprep Spin Miniprep kit. Restriction, modification, and ligation were carried out according to the manufacturer’s recommendations. DNA fragments were amplified in a Hybaid PCR thermocycler using Phusion DNA polymerase (NEB, Beijing, China). Purification of DNA fragments from the PCR and restriction-digest reactions was performed using the TianGEN Extract II kit (TIANGEN, Beijing, China). The validity of all the fragments amplified by PCR was determined by sequencing (BGI, Guangzhou, China).

### Construction of *R*. *anatipestifer tonB* Deletion Mutants

The *tonB* genes were deleted by allelic exchange using a recombinant suicide vector, which replaced *tonB* genes with a 1140-bp SpcR cassette according to the methods described by Hu et al [37] or a 994-bp ErmR cassette from *R*. *anatipestifer* CH-1. Briefly, the approximately 800-bp left flanking sequence and 800-bp right flanking sequence of the *R*. *anatipestifer* ATCC11845 *tonB1* and *tonB2* genes were amplified by PCR using primers TonB1upP1 and TonB1upP2, TonB1downP1 and TonB1downP2, TonB2upP1 and TonB2upP2, TonB2downP1 and TonB2downP2, respectively (S1 Table). The 1140-bp sequence containing the SpcR cassette was amplified from plasmid pAM238 using primers SpcRP1 and SpcRP2. The 994-bp sequence containing the ErmR cassette was amplified from the genome of *R*. *anatipestifer* CH-1 using primers ErmRP1 and ErmRP2. The PCR fragments (TonB1 upstream, TonB1 downstream, and ErmR cassette or TonB2 upstream, TonB2 downstream, and SpcR cassette) were ligated using the overlap PCR method [38]. The fused PCR fragments were purified and digested with KpnI and BamHI. Then, fragments were ligated into a suicide pEX18Gm plasmid digested with KpnI and BamHI. Ligation mixtures were transformed into CaCl_2_-competent *E*. *coli* strain XL1-Blue cells and transformants were selected on LB plates containing Gen (20 μg/ml final concentration). Clones were screened by PCR using ErmR cassette primers or SpcR cassette primers. Two PCR-positive clones were then sequenced.

The donor strain *E*. *coli* S17-1, harboring the suicide vector pEX18Gm with the insert, and the recipient strain RA ATCC11845 were grown on LB or sheep blood plates, respectively, at 37°C overnight. Samples of the donor strain and the recipient strain were washed three times using 10 mM MgSO_4_. Then, the donor strain and the recipient strain were mixed at a ratio of 1:2 (2.5×10^8^: 5×10^8^), and filtered through a Millipore membrane. The membrane was incubated on a blood agar plate at 30°C for 8 h. The transconjugants were selected on blood agar plates supplemented with Kan (20 μg/ml) and Erm (1 μg/ml) or Spc (80 μg/ml). The gene-deletion mutant strains were identified by PCR by amplifying the conserved 16S rRNA gene of *R*. *anatipestifer* using primers 16SrRNAP1 and 16SrRNAP1 and the deleted gene using the corresponding cloning primers (TonB1P1 and TonB1P2, TonB2P1 and TonB2P2). To exclude the possibility that gene inactivation had a polar effect on transcription of adjacent genes, total RNA was isolated from the mutant and wild-type (Qiagen), and RT-PCR (Qiagen) was performed to measure mRNA levels of the downstream gene (S1 Fig).

### Construction of the Recombinant Vector for Complementation and Expression

Complete *R*. *anatipestifer tonB1*, *tonB2*, *tonB3*, *exbBDtonB1*, *exbBDDtonB2* and *OMhemR*_*RA*_ genes were amplified by PCR from *R*. *anatipestifer* chromosomal DNA using primers TonB1P1 and TonB1P2, TonB2P1 and TonB2P2, TonB3P1 and TonB3P2, ExbB1P1 and TonB1P2, ExbB2P1 and TonB2P2, OMhemRP1 and OMhemRP2, respectively (S1 Table), for complementation. Complete *R*. *anatipestifer tonB1*, *tonB2*, and *tonB3* genes were amplified by PCR from *R*. *anatipestifer* chromosomal DNA using primers TonB1P1 and TonB1P2 his, TonB2P1 and TonB2P2 his, TonB3P1 and TonB3P2 his, respectively (S1 Table), for expression of TonB proteins. These PCR fragments were purified and digested with EcoRI and HindIII except for ExbBDDTonB2, which was digested with NheI and HindIII. Then, fragments, except for the *OMhemR* fragment, were ligated into the pBAD24 plasmid digested with corresponding restriction endonucleases. *OMhemR* fragment was ligated into the pAM238 plasmid digested with the corresponding restriction endonucleases. Ligation mixtures were introduced into the CaCl_2_-competent *E*. *coli* XL1-Blue strain and transformants were selected on LB plates containing Amp at 100 μg/ml. Clones were screened by the PCR method with corresponding primers. Validity of the sequences was determined by sequencing.

### Expression and Purification of Recombinant His-tagged TonB proteins

Strains JP313 pBAD24::*tonB1*, JP313 pBAD24::*tonB2* and JP313 pBAD24::*tonB3* were grown overnight at 37°C in LB broth containing 100 μg/ml Amp. Then, 200 ml LB broth containing 100 μg/ml Amp were inoculated to an OD600 of 0.05 with the overnight culture and grown at 37°C. Expression was induced at an OD_600_ of 0.6 for 2 h by adding 0.02% arabinose. Bacteria were harvested by centrifugation for 10 min at 5,000 g and 4°C, and the pellet was suspended in 20 ml binding buffer (50 mM Tris-HCl, 250 mM NaCl, 0.05% triton, pH 8.0) containing lysozyme (1 mg/ml final concentration) and DNaseI (1 U/ml final concentration). Lysis of bacteria was obtained by freezing and thawing at least three times. The suspension was then centrifuged at 12,000 rpm for 30 min at 4°C. The supernatant containing the soluble fraction was mixed with 200 μl of Ni-NTA-agarose beads according to the manufacturer’s instructions. Purified protein was dialyzed twice against a buffer containing 50 mM Tris-HCl to eliminate any residual imidazole. The protein was stable for at least one month when kept at -80°C with 20% glycerol.

### Antibody Preparation

200 μl of an emulsion containing each purified TonB (50 μg) and Freund’s adjuvant (100 μl) were inoculated twice (at half-month intervals) into 3 four-week-old KunMing mice obtained from the Chengdu DaShuo Biological Technology Co., LTD. Two weeks after the second inoculation, 200-μl blood samples were collected every 3 weeks via retro-orbital bleeding when the mice were anesthetized from smelling ether. At the end of the study, the mice were euthanized with 150 mg/kg sodium pentobarbital. Blood samples were centrifuged twice (3,600 rpm for 5 min) to obtain serum, which was stored at -20°C. Before use, non-specific antibodies were removed by incubating the immune serum with *E*. *coli* cell extract for 1 h at 4°C followed by centrifugation for 10 min at 8,000 rpm. The supernatant was then used as serum.

### Protein Analysis by Electrophoresis

Proteins were analyzed by 12% sodium dodecyl sulfate polyacrylamide gel electrophoresis (SDS-PAGE) followed by Coomassie Blue staining.

### Immunoblot Analysis

SDS-PAGE and immunoblotting, used to detect expression of TonB in *R*. *anatipestifer* or *E*. *coli* were performed as follows. The biomass of tested strains was collected, suspended in PBS buffer, and centrifuged. Bacterial pellets calculated to contain 20 μg of protein were suspended in loading buffer and heated for 5 min at 100°C. Proteins were separated by 12% SDS-PAGE and subsequently transferred to a nitrocellulose membrane. Non-specific binding sites were blocked with 5% skim milk in TBS-Tween 20 (0.05%). The blot was probed with polyclonal mouse sera raised against recombinant TonB (1:400), followed by a 1:2,000 dilution of a goat anti-mouse IgG alkaline phosphatase-conjugated secondary antibody (CST). The binding of antibodies to TonB protein was revealed using BCIP/NBT solution following the manufacturer’s instructions (Sigma).

### Protein Assay

The concentrations of the TonB proteins were determined using the BCA assay protein quantitation kit (Thermo Fisher).

### Statistical Analysis

Statistical analysis was performed using GraphPad Prism 5 software for Windows. Statistical significance of the data was ascertained by use of Student’s T test. A value of P<0.05 was considered significant.

### Ethics Statement

Animals were handled in strict accordance with good animal practice as defined by the local animal welfare bodies. Animal work performed at the Sichuan Agriculture University was reviewed and approved by the Sichuan Agriculture University ethics committee in September 2014.

## Results

### Sequence Analysis of the TonB Genes from *R*. *anatipestifer*

**Fig 1:**
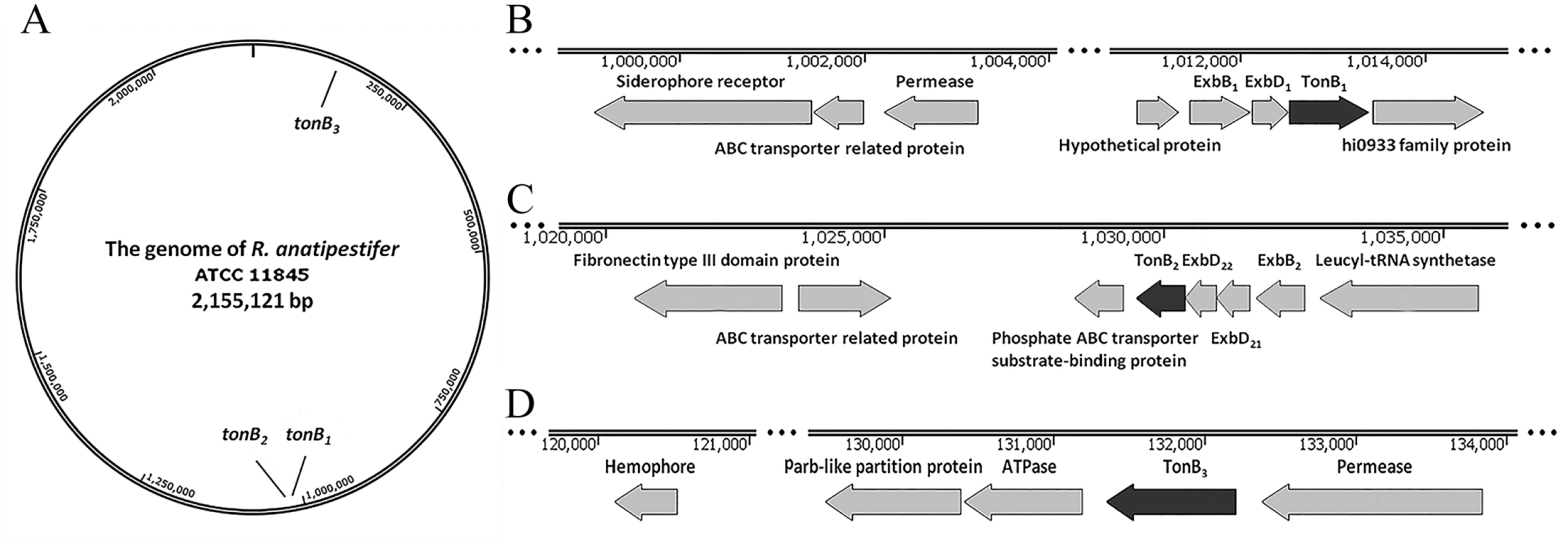
Genetic maps of *R*. *anatipestifer* ATCC11845 TonB systems. Shown are the locations of the three *tonB* loci in the ATCC11845 chromosome (A) and the genetic organization within the ATCC11845 DNA regions, including the *tonB1* (B), *tonB2* (C) and *tonB3* (D) genes and nearby related genes. DNA positions in the chromosome are given by the number at the top of each panel. Each horizontal arrow indicates the location of the coding region, and the direction of transcription for each *tonB* gene. The name of each gene is shown above or below the arrow.

Genome analysis revealed the locations of three hypothetical *tonB* genes in the *R*. *anatipestifer* genome (Fig 1A). We termed them *tonB1*, *tonB2* and *tonB3*, respectively. The *tonB1* and *tonB2* genes are organized into operons with their corresponding *exbB* and *exbD* genes, similar to the organization in *Campylobacter jejuni*, *Vibrio anguillarum* and *Vibrio alginolyticus* [39–41]. In contrast to *tonB1*, the *tonB2* operon includes two corresponding *exbD* genes, as occurs in *Flavobacterium psychrophilum*, *Acinetobacter baumannii* and *Xanthomonas campestris* [34,42,43]. The *tonB3* is present as a monocistronic copy, like in *Acinetobacter baumannii* [43], which does not correspond with expectations based on the *exbB* and *exbD* genes in *R*. *anatipestifer*.

The first hypothetical TonB1 system contains three genes: *exbB*, coding for a 24-kDa protein, *exbD*, coding for a 14.7-kDa protein, *tonB*, coding for a 30.6-kDa protein. This system is flanked by an upstream gene transcribed in the same direction that codes for a hypothetical protein and a downstream gene transcribed in the same direction that codes for a predicted hi0933 family protein (Fig 1B). It is worth noting that approximately 8 kb upstream of TonB1 system, there are three related genes transcribed in the opposite direction that code for a TonB-dependent siderophore receptor, an ABC transporter related protein, and a transport-system permease protein. The second hypothetical TonB2 system contains four genes: *exbB2*, coding for a 30.8-kDa protein, *exbD21*, coding for a 21.1-kDa protein, *exbD22*, coding for a 20.4-kDa protein, and *tonB2*, coding for a 31-kDa protein. The TonB2 system, which is located 16 kb downstream of the TonB1 system, is flanked by an upstream gene transcribed in the same direction that codes for a leucyl-tRNA synthetase and a downstream gene transcribed in the same direction that codes for an phosphate ABC transporter substrate-binding protein (Fig 1C). In addition, genes coding for an ABC transport related protein and fibronectin, which may have some connection with TonB [44], are closely located to the TonB2 system. The third *tonB* gene, coding for a 31-kDa protein, is located far apart from the other two *tonB* gene clusters and is flanked by two genes that code for a permease-family protein and a ATPase (Fig 1D). The analysis of different *R*. *anatipestifer* strains, including ATCC, CH-1, and CH-2, showed that the TonB systems have similar organizational styles in all sequenced genomes and high nucleotide sequence similarity (more than 90%, data not shown)

In *A*. *baumannii*, the three TonB proteins have high sequence identity to each other [43]. However, in *R*. *anatipestifer*, there is only approximately 10.00% identity between TonB1 and TonB2, 10.65% identity between TonB1 and TonB3, and 34.71% identity between TonB2 and TonB3. Their transmembrane domains also have a low identity, less than 17% (data not shown). A similar degree of divergence was observed in ExbB, less than 22% identity (data not shown). However, the transmembrane domains of ExbD1 and ExbD22 have 43.3% identity (data not shown). Sequence analysis showed that the closest homologs of these three TonB proteins are mainly distributed in the *Flavobacteria*, *Cytophagia*, *Sphingobacteria* and *Bacteroidia* classes and show greater than 25% identity.

**Fig 2:**
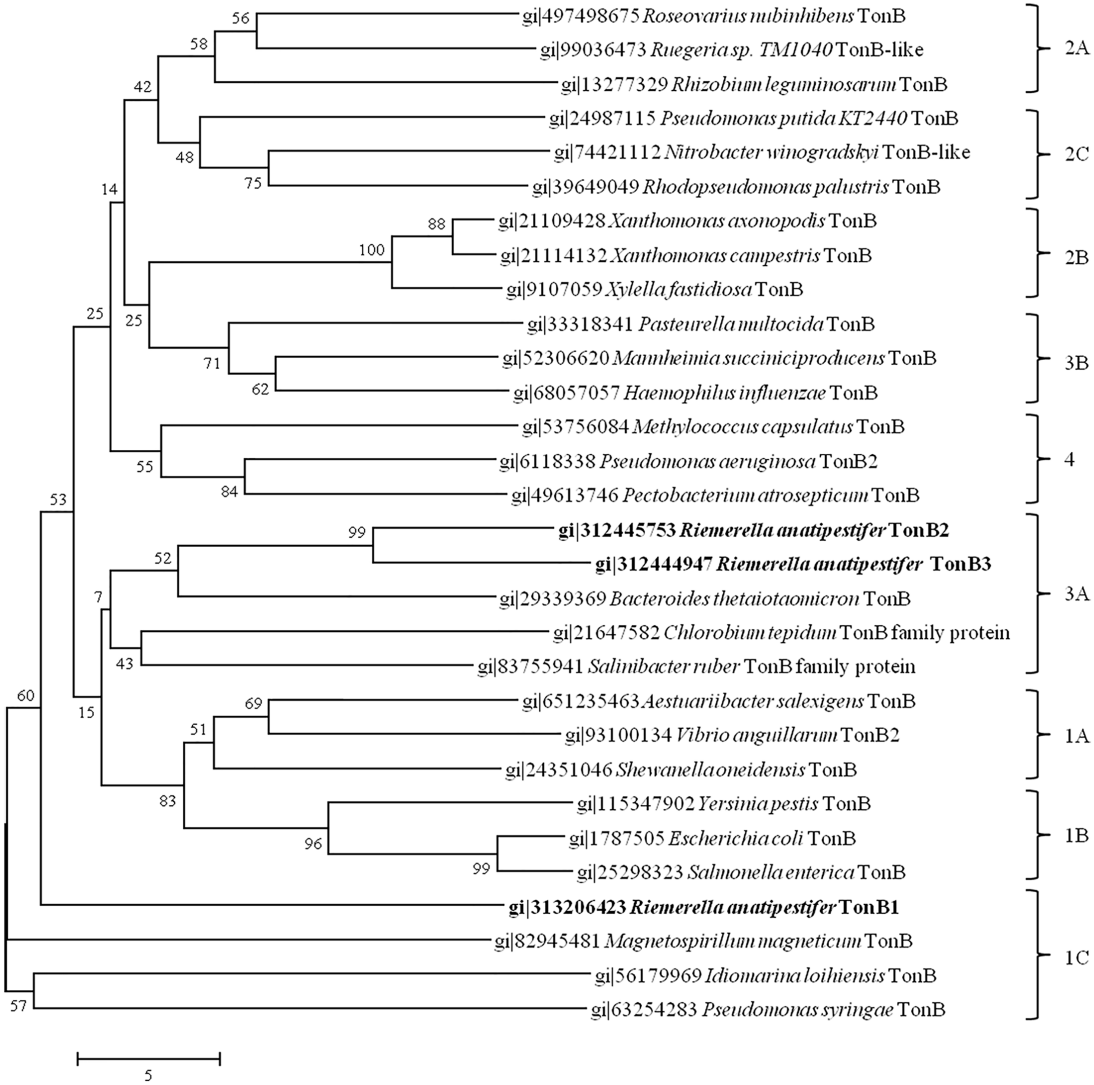
Phylogenetic tree of TonB C-terminal domains. The tree was created using a Neighbor-Joining Bootstrap method with 1000 bootstrap trials in ClustalW derived from the MSA shown in S2 Fig. The trees were drawn and visualized with MEGA v6.05. The clusters (1A–C, 2A–C, 3A–B and 4 shown on the right) were generated by previous research [27]. TonB proteins from *R*. *anatipestifer* are shown with overstriking typeface.

The carboxy-terminal domain (CTD) of TonB proteins is believed to be essential for interactions with the TonB-dependent outer membrane receptor TonB-box [5,45]. Therefore, studies of the CTD of TonB are important for the further understanding for the mechanism of TonB energy support for hemin or iron acquisition. Based on the ClustalW-based multiple sequence alignment (MSA) (S2 Fig) for representative sequences from each of the nine CTD clusters of TonB proteins from gram-negative bacteria, as classified by Byron [27], and the three TonB proteins of *R*. *anatipestifer*, the neighbor-joining bootstrap tree (Fig 2) showed the evolutionary relationship between the CTDs of the three *R*. *anatipestifer* TonBs with other representative TonBs from previous studies [27]. The result indicates that the CTDs of TonB2 and TonB3 fall into the 3A cluster while that of TonB1 falls into the 1C cluster, where 3A and 1C refer to a classification system developed by Byron to distinguish CTDs from TonB proteins [27]. Similarly, the secondary structure of the TonB2 CTD is almost the same as that of the TonB3 CTD, while the secondary structure of the TonB1 CTD is clearly different (data not shown). These results indicate that TonB2 and TonB3 are similar in sequence and structure. However, the three TonB proteins of *R*. *anatipestifer* show the differences between the CTDs of each TonB protein (S2 Fig). All three TonBs lack the highly conserved YP motif (S2 Fig), which has been identified in close contact with the TonB box of TonB-dependent transporters [46,47]. Moreover, we were unable to identify the highly conserved SSG motif, which has been identified in many different TonB sequences and is believed to play a role in outer-membrane receptor recognition [27].

### Both TonB1 and TonB2 Systems Are Required for Hemin Uptake in *R*. *anatipestifer*

To investigate the roles of the TonB systems of *R*. *anatipestifer*, we first tried to construct the isogenic derivatives of *R*. *anatipestifer* ATCC11845 deleted for each TonB system. We successfully built mutants deleted for *tonB1* or *tonB2*, and a mutant deleted for both *tonB1* and *tonB2*. Validation of the mutants was performed by PCR and western-bolt experiments using antibodies directed against TonB1 or TonB2 (S3 Fig). However, we did not successfully build a *tonB3* mutant despite many attempts. A similar situation was also observed in *A*. *baumannii* [43], and we hypothesize that TonB3 is an essential gene for *R*. *anatipestifer* ATCC11845.

**Fig 3:**
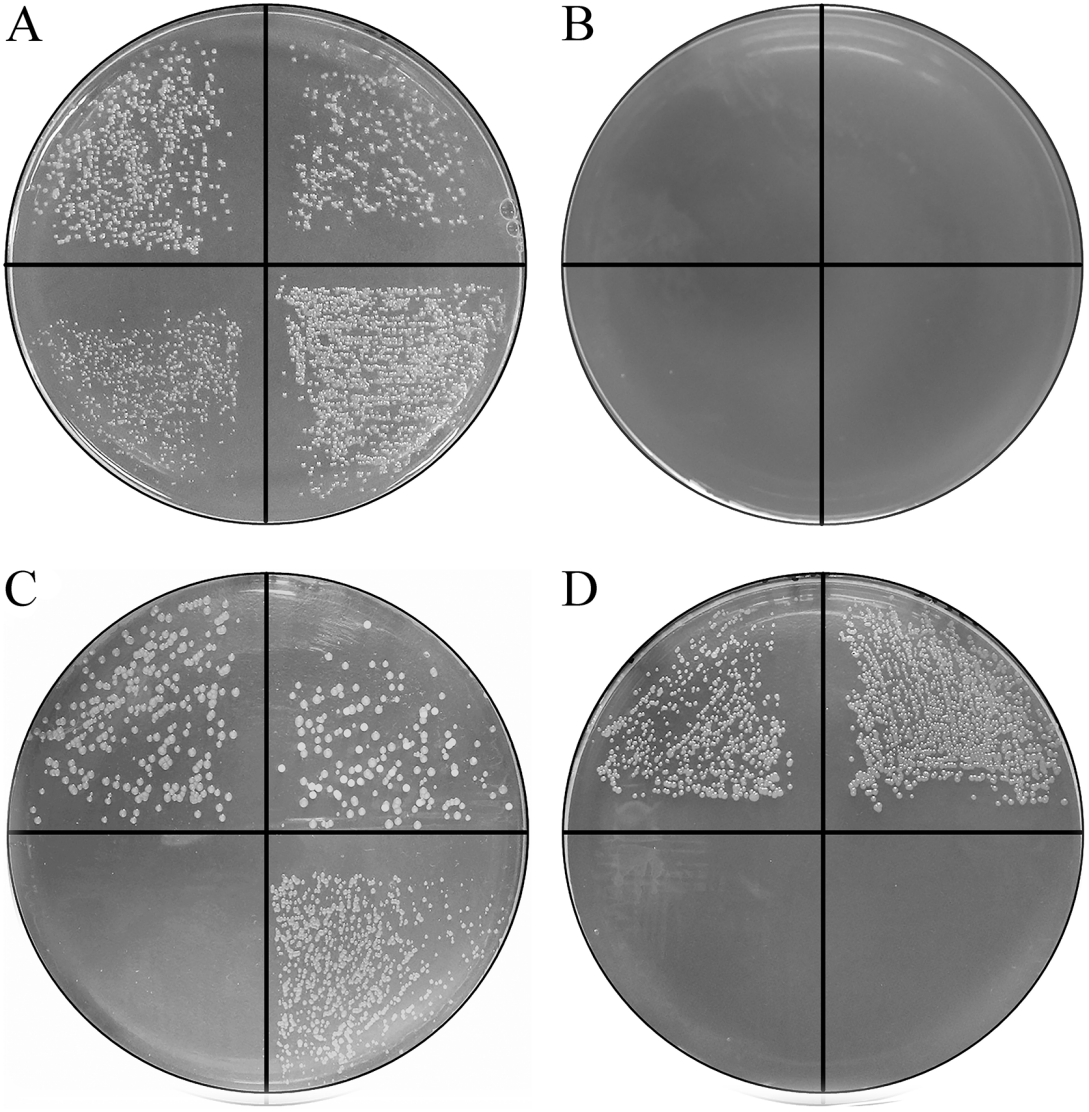
The effect of *R*. *anatipestifer* TonB knockouts on hemin and iron utilization. *R*. *anatipestifer* strains (clockwise from the top left) ATCC11845, *tonB1* mutant, *tonB2* mutant and *tonB1tonB2* double-mutant were spread on LB plates containing 5% bovine serum (A), LB-only plates (B), LB plates containing 20 μM hemin (C), and LB plates containing 5% bovine serum and 40 μM Dip (D). Growth was assessed by appearance of bacterial colonies on the plates. Pictures were taken after 48 hours of growth at 37°C under 5% CO_2_ atmosphere. All the experiments were repeated three times. A representative result is presented.

*R*. *anatipestifer* ATCC11845 is unable to grow on LB plates because of the lack of the essential hemin synthesis genes *hemF*, *hemY*, and *hemG* [4]. However, *R*. *anatipestifer* ATCC11845 can grow on LB plates containing serum (Fig 3A), which is hypothesized to provide hemin. To demonstrate that hemin is essential for *R*. *anatipestifer* ATCC11845, we first checked the effect of hemin addition on bacterial growth on LB medium. The results showed that *R*. *anatipestifer* ATCC11845 was able to grow on LB plates only in the presence of 20 μM hemin (Fig 3B and 3C). Surprisingly, other commonly used hemin sources like bovine hemoglobin were insufficient to support the growth of *R*. *anatipestifer* ATCC11845 on LB medium (data not shown). To identify whether the TonB systems were involved in hemin uptake, we checked for the growth of *R*. *anatipestifer* ATCC11845 *tonB1* and *tonB2* mutants on LB plates in the absence and presence 20 μM hemin. Our results showed that the wild-type strain and *tonB1* mutant strain formed small colonies after 24 h growth in the presence of hemin. In contrast, no growth was observed for the *tonB2* mutant or *tonB1tonB2* double mutant (data not shown). After 48 hours incubation, the *tonB2* mutant strain gradually formed pinpoint colonies. In contrast, the *tonB1tonB2* double mutant strain failed to exhibit any growth after 48 hours of incubation (Fig 3C). All these strains could be grown on LB plates containing 5% bovine serum and had no growth on LB-only plates (Fig 3A and 3B).

### The Fe^3+^ Acquisition of *R*. *anatipestifer* is Mainly Supported by the TonB2 System

To identify the TonB systems of *R*. *anatipestifer* involved in iron uptake, we tested *R*. *anatipestifer tonB* mutants for the growth on iron-chelated medium. The results showed that all the strains grew well on LB plates containing 5% bovine serum (Fig 3A). The wild-type strain and *tonB1* mutant grew well on plates supplemented with 40 μM Dip. In contrast, the *tonB2* mutant and *tonB1tonB2* double mutant grew poorly on the same medium (Fig 3D). These results suggested that the TonB2 system of *R*. *anatipestifer* plays an important role in iron uptake.

### Activity of TonB Systems of *R*. *anatipestifer* Expressed in *E*. *coli*

As described above, TonB2 is involved in iron and hemin uptake processes in *R*. *anatipestifer*. *TonB1* disruption strengthens the effect of the *tonB2* mutation on hemin uptake. A heterologous host like *E*. *coli* is commonly used to characterize the activity of TonB proteins from various gram-negative bacteria, such as *Bartonella birtlesii* [48] and *A*. *baumannii* [43]. To characterize the function of the three TonB systems from *R*. *anatipestifer*, we assessed the ability of each system to complement a *tonB* mutation in *E*. *coli*. We thus compared the ability of the three TonB proteins and two TonB complexes from *R*. *anatipestifer* to provide energy to a heterologous heme transporter, HasR or HemR from *S*. *marcescens* [31], expressed in an *E*. *coli tonB* mutant. We introduced plasmids expressing TonB proteins from *R*. *anatipestifer* into the *E*. *coli* strain C600*ΔhemA tonB*::Tn*10* pAM*238*::*hasR*/*hemR* and checked the growth on LB plates in the presence of hemin. All the C600*ΔhemA tonB*::Tn*10* pAM*238*::*hasR*/*hemR* derivative strains, producing TonB1 complex, TonB2 complex or TonB3 protein from *R*. *anatipestifer* (S4 Fig), were unable to grow in the presence of hemin (data not shown).

**Fig 4:**
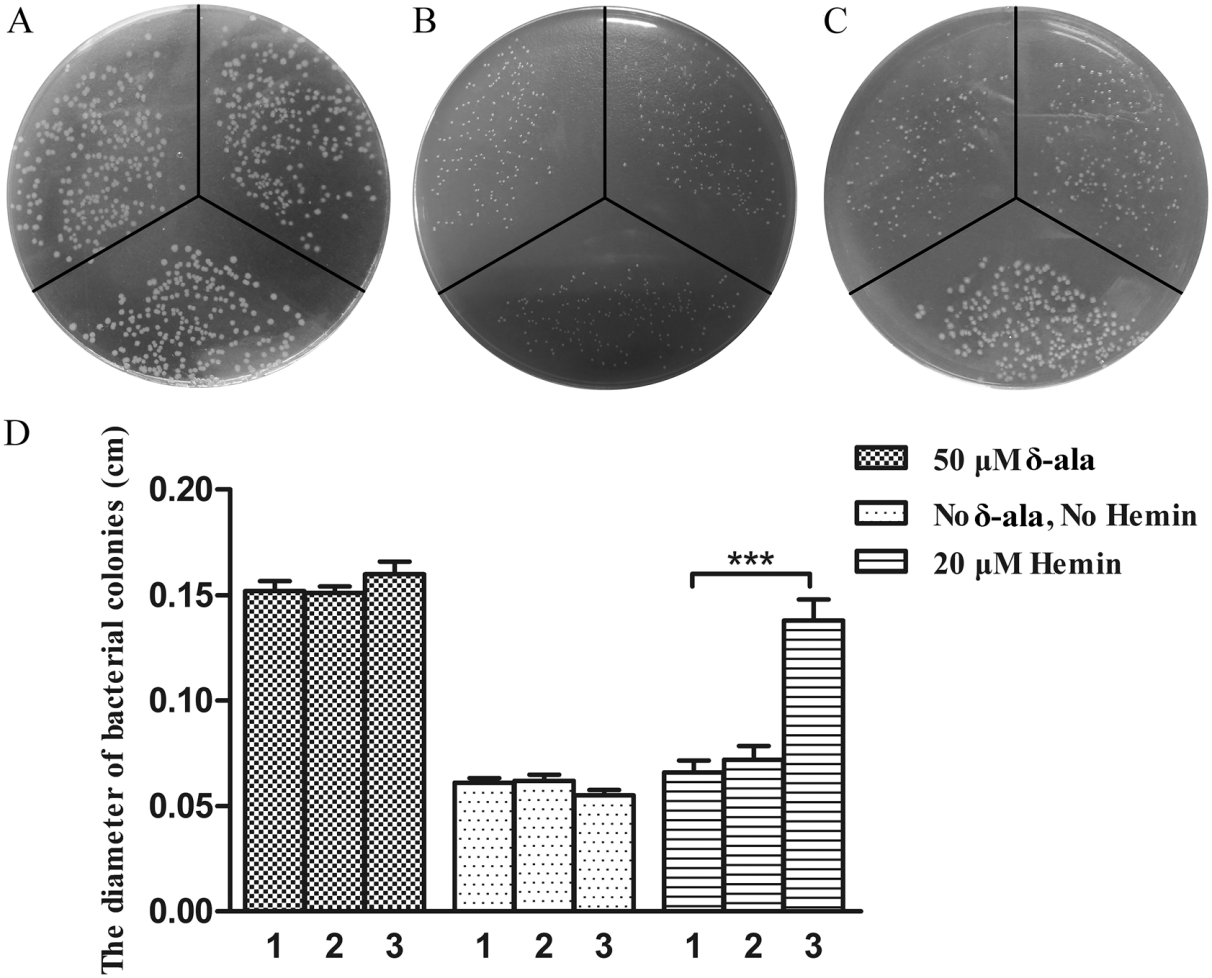
Functional complementation of the *E*. *coli tonB* mutant impaired in hemin uptake. *E*. *coli* strains (clockwise from the top left) C600*ΔhemA tonB*::Tn*10* pAM238::*OMhemR*_*RA*_ harboring (1) pBAD24, (2) pBAD24::*tonB1* and (3) pBAD24::*exbB1-exbD1-tonB1* were inoculated on LB plates containing 50 μg/ml δ-ala (A), LB-only plates (B) and LB plate containing 20 μM hemin (C). Fig 4D shows the diameter of the different *E*. *coli* colonies on these plates. Three asterisks indicate significant differences (P<0.001). Error bars represent standard errors from measurements of ten bacteria colonies. All the experiments were repeated three times. A representative result is presented.

The energizing activity of the three TonB proteins was also investigated in a *E*. *coli* C600*ΔhemA tonB*::Tn*10* derivative expressing a putative TonB-dependent hemin transporter from *R*. *anatipestifer* (gene ID: 11995802) cloned into plasmid pAM238 (pAM238::*OMhemR*_*RA*_). First, the plasmid pBAD24 and its derivatives expressing TonB1, TonB2,TonB3 or TonB2 complex were introduced in strain C600*ΔhemA tonB*::Tn*10* pAM238::*OMhemR*_*RA*_ and the resulting strains were tested for growth in the absence or in the presence of hemin. All the tested strains grew well on LB plate supplemented with 50 μM δ-ala (Fig 4A) while they showed limited growth on LB plates or LB plates containing 20 μM hemin (data not shown). In contrast, the C600*ΔhemA tonB*::Tn*10* pAM238::*OMhemR*_*RA*_ derivative strain expressing ExbB-ExbD-TonB1 of *R*. *anatipestifer* showed limited growth on LB plates (Fig 4B) and grew well on LB plates containing 20 μM hemin (Fig 4C and 4D). These results indicate that TonB1 cannot interact with ExbB-ExbD from *E*. *coli* and that only the complete ExbB1-ExbD1-TonB1complex from *R*. *anatipestifer* can energize OMHemR_RA_ for its hemin uptake activity.

### The TonB1 System of *R*. *anatipestifer* can Restore the Growth of C600*ΔhemA tonB*::Tn*10* under Iron-chelated Conditions

**Fig 5:**
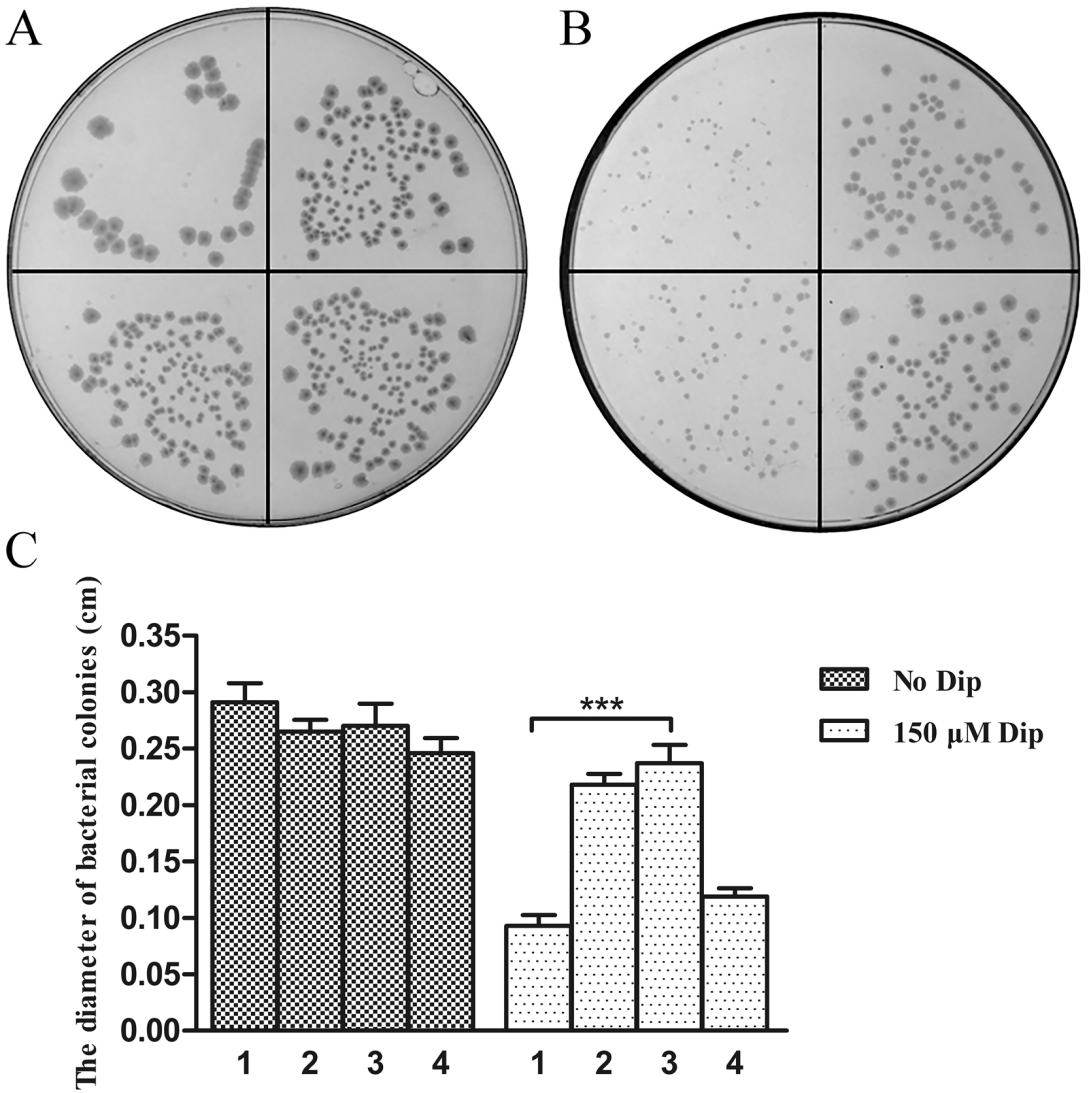
Functional complementation of the *tonB* mutant *E*. *coli* impaired in iron uptake. *E*. *coli* strains (clockwise from the top left) including (1) C600*ΔhemA tonB*::Tn*10* pBAD24, (2) C600*ΔhemA* pBAD24, (3) C600*ΔhemA tonB*::Tn*10* pBAD24 *exbB*-*exbD*-*tonB1* and (4) C600*ΔhemA tonB*::Tn*10* pBAD24::*tonB1* were grown on LB plates containing δ-ala (50 μg/ml), Amp (100 μg/ml) and 0 μM Dip (A) or 150 μM Dip (B). Part C shows the diameter of the *E*. *coli* colonies on the plates. Three asterisks indicate significant differences (P<0.001). Error bars represent standard errors from measurements of ten bacteria colonies. All the experiments were repeated three times. A representative result is presented.

In *E*. *coli*, all the iron Fe^3+^ uptake systems require the energizing activity of TonB. An *E*. *coli tonB* mutant cannot grow on iron-depleted media. We assessed the ability of TonB1 or the ExbB1-ExbD1-TonB1 system of *R*. *anatipestifer* to energize *E*. *coli* iron transporters. The C600*ΔhemA tonB*::Tn*10* strain derivatives trains expressing TonB1 or ExbB1-ExbD1-TonB1 from *R*. *anatipestifer* were tested for growth on LB plates with δ-ala in the absence or in the presence of 150 μM iron chelator Dip. The tested strains grew as well as the positive control C600*ΔhemA* pBAD24 on LB δ-ala plates in the absence of Dip (Fig 5A). Addition of Dip strongly inhibited the growth of strains C600*ΔhemA tonB*::Tn*10* pBAD24 and C600*ΔhemA tonB*::Tn*10* pBAD24::*tonB1* (Fig 5B). In contrast, expression of the complete ExbB1-ExbD1-TonB1 system in the strain allowed normal growth of the C600*ΔhemA tonB*::Tn*10* pBAD24::*exbB1-exbD1-tonB1* strain in the presence of Dip, compared with strain C600*ΔhemA* pBAD24 (Fig 5B and 5C).

We also checked for the ability of TonB2, ExbB2-ExbDD-TonB2 and TonB3 to restore the growth of C600*ΔhemA tonB*::Tn*10* derivative strains in the presence of Dip. The results showed that the strain expressing TonB2, ExbB2-ExbDD-TonB2 and TonB3 had no increase in growth compared to the negative control C600*ΔhemA tonB*::Tn*10* pBAD24 under iron-limited conditions (data not shown).

## Discussion

*R*. *anatipestifer* does not encode the genes *hemF*, *hemY*, and *hemG*, which are essential for *de novo* synthesis of hemin. Hemin is not only an essential component as the prosthetic group of many proteins [4], but it is also a major iron source [8]. TbdR1 was shown to be a potential outer-membrane hemin receptor and hemin was shown to act as a potential iron source in *R*. *anatipestifer* [8], indicating that *R*. *anatipestifer* has the ability to express at least one hemin uptake system that includes a TonB system. Previous research revealed that the transport of siderophore-loaded Fe^3+^ is also TonB dependent due to the energy transducing roles of TonB [8,9]. In this study, we proved that TonB proteins in *R*. *anatipestifer* are required for both iron and hemin transport.

*R*. *anatipestifer* encodes two sets of TonB systems and one TonB family protein. The TonB system involved in hemin acquisition and/or iron uptake cannot be predicted *in silico* because analysis of the neighborhood of genes encoding these three TonB systems do not clearly suggest the individual roles of the three TonB proteins in hemin or iron uptake. For many gram-negative bacteria, such as *P*. *damselae* [49] and *V*. *anguillarum* [50], hemin/iron uptake-related genes are located in the same operon as the *tonB* gene in a cotranscribed direction. For other gram-negative bacteria, such as the *Flavobacteriia sychrophilum* [34], *tonB* genes are not located next to other genes encoding hemin/iron uptake systems. In still other types of gram-negative bacteria, such as *Serratia marcescens*, *one tonB-encoding gene is located in a transcription unit expressing its cognate heme uptake system*, *and the other tonB* neighborhood not contains genes encoding for hemin or iron uptake systems [31].

To explore the function of TonB systems in hemin and iron acquisition in *R*. *anatipestifer*, we sought to construct isogenic *tonB* mutant strains. We used spectinomycin-resistance genes to replace *tonB* genes as described by Hu et al [37]. However, we found that the single *tonB* mutants did not produce a strong growth defect on the hemin plates or iron-depleted plates. Therefore, we constructed the double mutant for the TonB1 and TonB2 systems of *R*. *anatipestifer*. Using sequence analysis, we found that the genome of *R*. *anatipestifer* CH-1 encodes for an erythromycin-resistance gene not present in the genome of *R*. *anatipestifer* ATCC11845. Therefore, we attempted to use this cassette to replace the *tonB* genes. Attempts were successful for the *tonB1* and *tonB2* genes, though we failed to construct a *tonB3* mutant strain using the spectinomycin or erythromycin cassettes. This result suggests that the activity of TonB3 is absolutely required for growth in *R*. *anatipestifer*. A similar situation was also observed for the *tonB* genes in *A*. *baumannii* [43]. Therefore, we supposed that *tonB3* may be an essential gene involved in fundamental nutrient acquisition.

Hemin transport is completely abolished only when both *tonB1* and *tonB2* are deleted. This result indicates that both TonB1 and TonB2 are involved in hemin transport in *R*. *anatipestifer* and that the proteins are able to compensate for one another. In addition, we demonstrated that TonB2 is more important than TonB1 for hemin uptake in *R*. *anatipestifer*.

To further investigate more precisely the role of TonB proteins of *R*. *anatipestifer* in hemin and iron acquisition, we assessed the capacity of the proteins to complement hemin utilization in *E*. *coli* model strain C600*ΔhemA tonB*::Tn10 pAM238::*hasR/hemR*. All the three *tonB* genes and two complete TonB system operons were insufficient to restore the growth of C600*ΔhemA tonB*::Tn10 pAM238::*hasR*/*hemR* under low-hemin conditions. This result reveals that the TonB systems of *R*. *anatipestifer* cannot work with the heterologous hemin transporter HasR/HemR of *S*. *marcescens* to transport hemin. This result can be explained by the very low polypeptide sequence identity (lower than 10%) between the three TonB proteins of *R*. *anatipestifer* and the TonB and HasB proteins of *S*. *marcescens*.

Our results obtained with *E*. *coli* demonstrate that the TonB1 complex of *R*. *anatipestifer* can energize OMhemR (AFD55772) of *R*. *anatipestifer*. That the TonB1 complex can energize OMHemR instead of HasR/HemR can be explained by the facts that OMhemR shares homologies lower than 10% identity with HasR/HemR (data not shown) and some TonB-dependent receptors function only with TonB from their original host [51]. This latter result strengthens the previous results suggesting that OMhemR (AFD55772) of *R*. *anatipestifer* is energized only by TonB1. Similar specificity of TonB systems toward a hemin transporter was also described in *S*. *marcescens* [31] and in *H*. *influenza* [52].

According to our results, the Fe^3+^ acquisition of *R*. *anatipestifer* is supported mainly by the TonB2 system. Surprisingly, in the *E*. *coli* model, only the TonB1 system from *R*. *anatipestifer* was capable of energizing the transport systems devoted to iron uptake. The question of why the TonB1 system can work with the Fe^3+^ receptor of *E*. *coli* but appears to be unnecessary for iron uptake in *R*. *anatipestifer* is not understood at this time. To identify receptors of *E*. *coli* worked with TonB1 system of *R*. *anatipestifer* is an interesting project for further study.

In view of the function of the TonB systems in hemin and iron acquisition in *R*. *anatipestifer*, we hypothesize that TonB systems influence the virulence of *R*. *anatipestifer*, similar to the cases observed in *S*. *dysenteriae* [53], *E*. *coli* [35] and *A*. *baumannii* [43]. Understanding the relationships between the individual TonB systems and the virulence of the respective bacteria is important for understanding the pathogenic mechanisms and for developing new ways to prevent *R*. *anatipestifer* infection.

## Supporting Information

S1 Fig The transcription of the downstream genes of *tonB1* and *tonB2* in wild type and mutant strains. Total RNA was isolated from the mutant and wild-type. Then, cDNA were obtained through reverse transcription and were acted as templates of RT-PCR. Total RNA were used as a control to exclude the contamination of DNA. Primers hi0933P1 and hi0933P2 (S1 Table) were used to amplify a 114 bp target in the downstream gene of *tonB1*. Primers ABCP1 and ABCP2 (S1 Table) were used to amplify a 154 bp target in the downstream gene of *tonB2*.
(TIF)

S2 Fig Alignment of TonB C-terminal domains. Representative sequences from each of the nine carboxy-terminal domain (CTD) clusters in gram-negative bacterial TonB proteins including the three TonB proteins from *R*. *anatipestifer* ATCC11845 (overstriking typeface) were subjected to multiple sequence alignment (MSA) by ClustalW. Regions are shaded based on the degree of similarity. The highly conserved YP and SSG motifs in most of the TonB proteins are shaded and marked with asterisks above the alignment.
(TIF)

S3 Fig Detection of *R*. *anatipestifer tonB* mutants by immunoblotting. Lanes 1 to 4 each contain 10 μg wild-type *R*. *anatipestifer*, *R*. *anatipestifer ΔtonB1*, *R*. *anatipestifer ΔtonB2*, *R*. *anatipestifer ΔtonB1 ΔtonB2* strains, respectively. TonB1 and TonB2 from top to bottom are probed with anti-TonB1 and anti-TonB2 serum and then detected by BCIP/NBT.
(TIF)

S4 Fig Detection of the expression of each TonB protein in *E*. *coli* by immunoblotting. Lane 1 in each panel (from top to bottom) contains 200 ng purified TonB1, TonB2 or TonB3, respectively. Lane 2 contains 10 μg *E*. *coli* strain C600*ΔhemA tonB*::Tn*10* pBAD24. Lane 3 from top to bottom contains 10 μg *E*. *coli* strains C600*ΔhemA tonB*::Tn*10* pBAD24::*tonB1*,:: *tonB2*, or:: *tonB3* induced by 0.02% arabinose, respectively. TonB1, TonB2 and TonB3 from top to bottom are probed with anti-TonB1, anti-TonB2 and anti-TonB3 serum and then detected by BCIP/NBT. All the experiments were repeated three times. A representative result is presented.
(TIF)

S1 Table Primers used in this study. (DOCX)
