# Supplementary figures and images for: TonB Energy Transduction Systems of Riemerella anatipestifer Are Required for Iron and Hemin Utilization
Source: PLoS One. 2015 May 27;10(5):e0127506. doi: 10.1371/journal.pone.0127506 (PMC4446302; doi:10.1371/journal.pone.0127506)

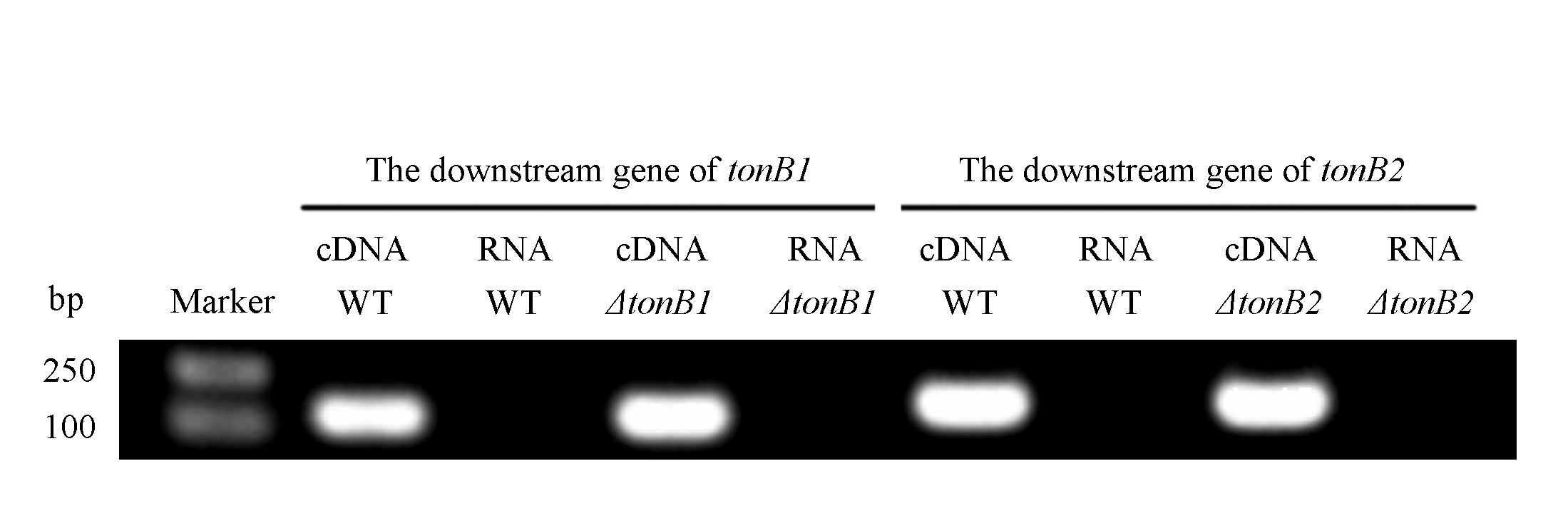

Supplement: S1 Fig — Total RNA was isolated from the mutant and wild-type. Then, cDNA were obtained through reverse transcription and were acted as templates of RT-PCR. Total RNA were used as a control to exclude the contamination of DNA. Primers hi0933P1 and hi0933P2 (S1 Table) were used to amplify a 114 bp target in the downstream gene of tonB1. Primers ABCP1 and ABCP2 (S1 Table) were used to amplify a 154 bp target in the downstream gene of tonB2. (TIF) [file pone.0127506.s001.tif]

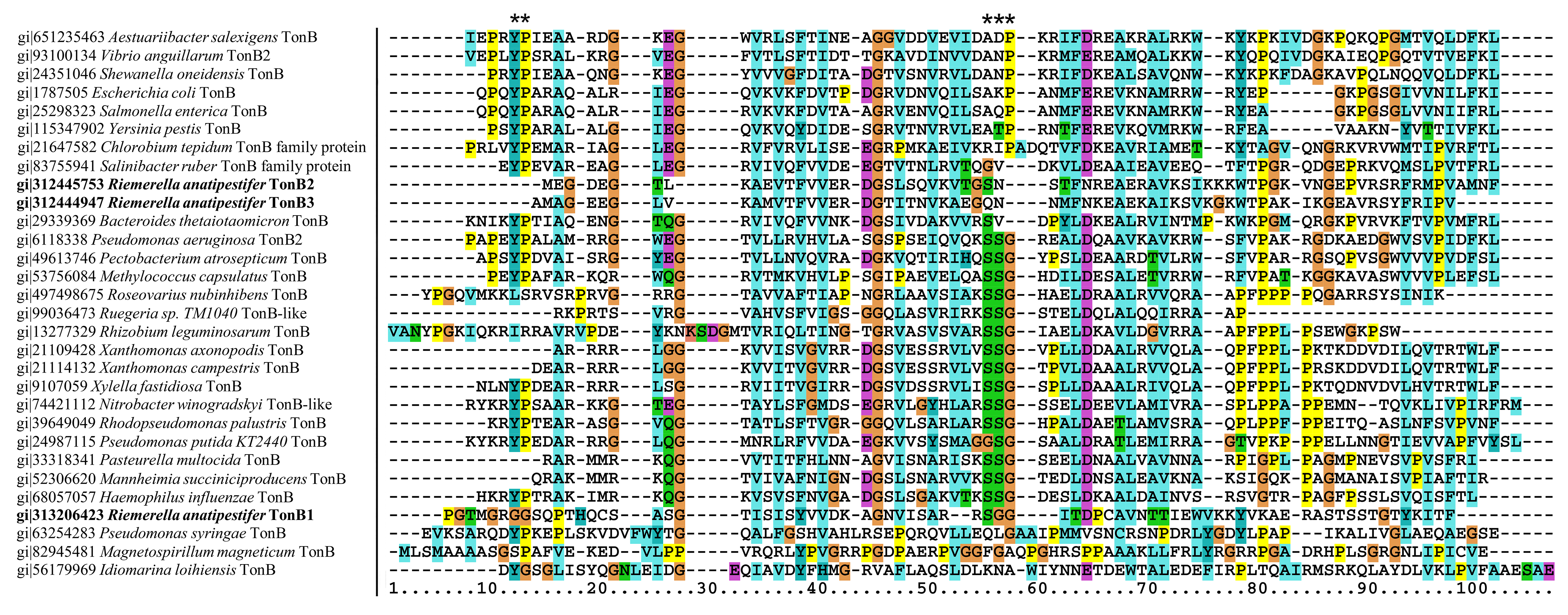

Supplement: S2 Fig — Representative sequences from each of the nine carboxy-terminal domain (CTD) clusters in gram-negative bacterial TonB proteins including the three TonB proteins from R. anatipestifer ATCC11845 (overstriking typeface) were subjected to multiple sequence alignment (MSA) by ClustalW. Regions are shaded based on the degree of similarity. The highly conserved YP and SSG motifs in most of the TonB proteins are shaded and marked with asterisks above the alignment. (TIF) [file pone.0127506.s002.tif]

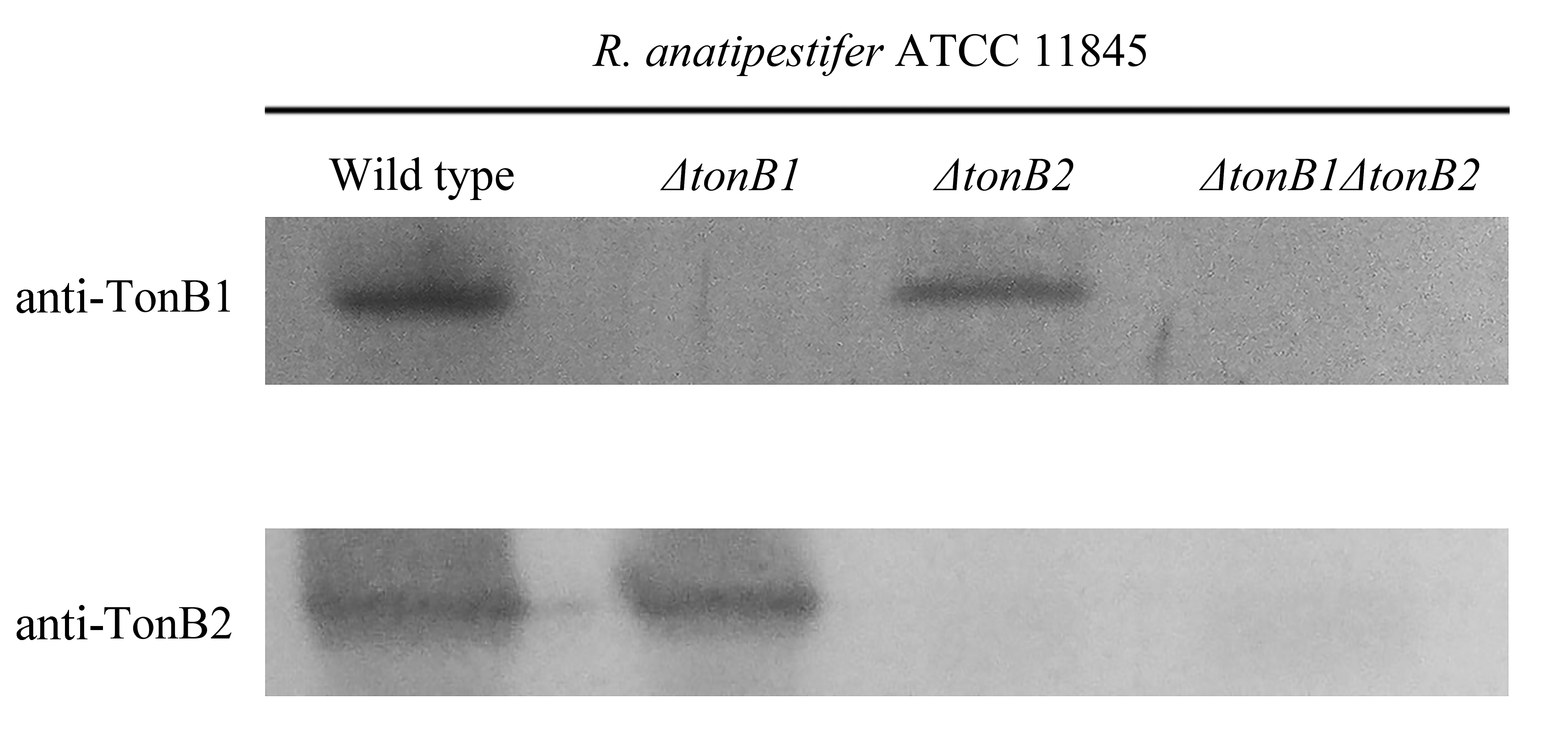

Supplement: S3 Fig — Lanes 1 to 4 each contain 10 μg wild-type R. anatipestifer, R. anatipestifer ΔtonB1, R. anatipestifer ΔtonB2, R. anatipestifer ΔtonB1 ΔtonB2 strains, respectively. TonB1 and TonB2 from top to bottom are probed with anti-TonB1 and anti-TonB2 serum and then detected by BCIP/NBT. (TIF) [file pone.0127506.s003.tif]

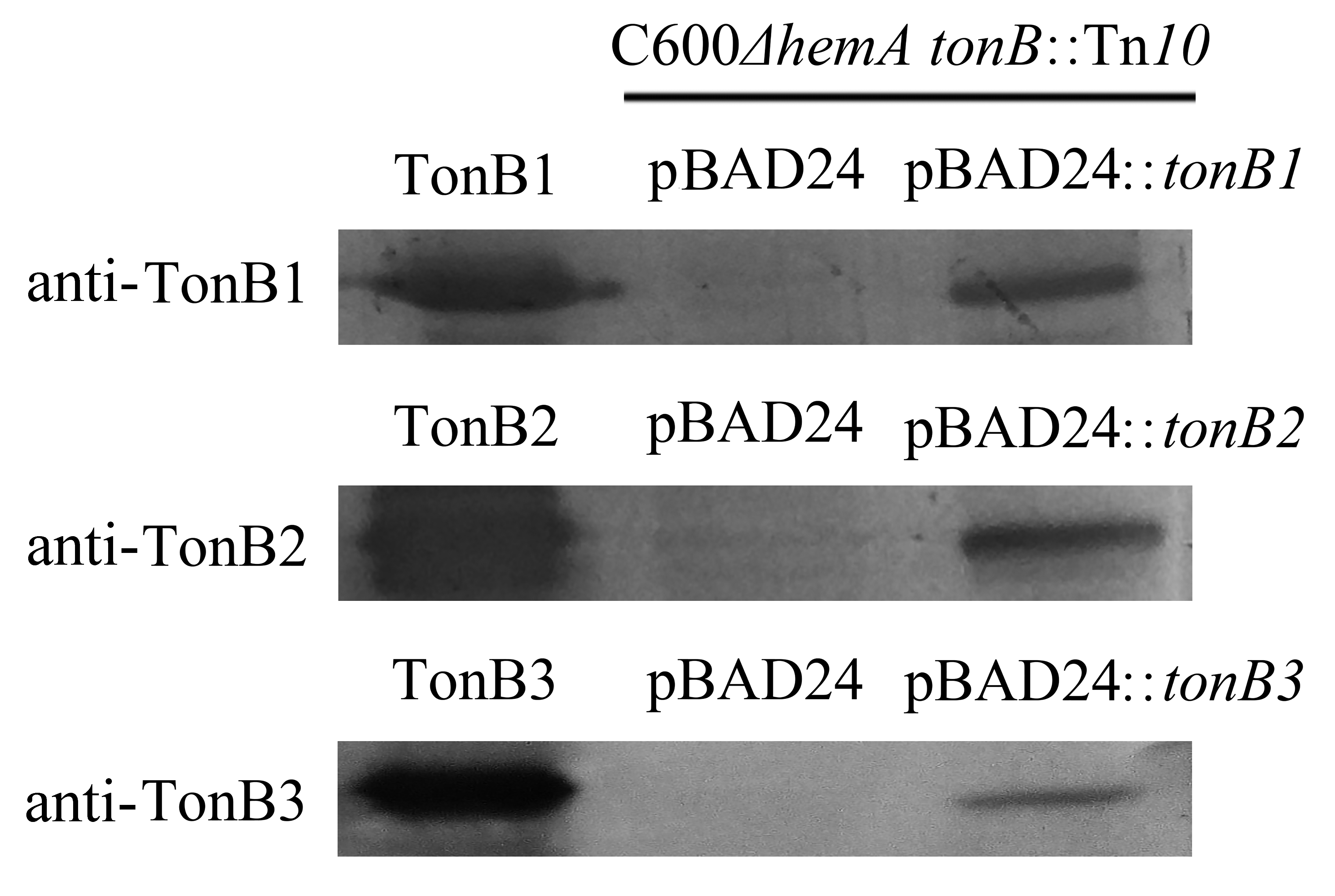

Supplement: S4 Fig — Lane 1 in each panel (from top to bottom) contains 200 ng purified TonB1, TonB2 or TonB3, respectively. Lane 2 contains 10 μg E. coli strain C600ΔhemA tonB::Tn10 pBAD24. Lane 3 from top to bottom contains 10 μg E. coli strains C600ΔhemA tonB::Tn10 pBAD24::tonB1,:: tonB2, or:: tonB3 induced by 0.02% arabinose, respectively. TonB1, TonB2 and TonB3 from top to bottom are probed with anti-TonB1, anti-TonB2 and anti-TonB3 serum and then detected by BCIP/NBT. All the experiments were repeated three times. A representative result is presented. (TIF) [file pone.0127506.s004.tif]
