# Supplementary material for: TonB Energy Transduction Systems of Riemerella anatipestifer Are Required for Iron and Hemin Utilization
Source: PLoS One. 2015 May 27;10(5):e0127506. doi: 10.1371/journal.pone.0127506 (PMC4446302; doi:10.1371/journal.pone.0127506)
Supplement: S1 Table — (DOCX) [file pone.0127506.s005.docx]

**Table S1. Primers used in this study.**

| **Primer** | **Organism** | **Sequence** |
| --- | --- | --- |
| TonB1P1 | *R. anatipestifer* ATCC11845 | AGGAGGAATTCACCATGAGCCAAACCATAAATACATCGG |
| TonB1P2 | *R. anatipestifer* ATCC11845 | ATCCCCGAAGCTTATGGTTTAGAAAGTGATTTTGTAAGTGCC |
| TonB2P1 | *R. anatipestifer* ATCC11845 | TTTTGGGCTAGCAGAGGAATTCACGTCATGTCAGATGAAAATTTAGG |
| TonB2P2 | *R. anatipestifer* ATCC11845 | CATAGGGTACCTCGGTGAAGCTTGGTGCTTAATACTCAAAATTCATTGCC |
| TonB3P1 | *R. anatipestifer* ATCC11845 | TTTGGGCTAGCAGGAGGATGATAACTTTTTCACAAAACC |
| TonB3P2 | *R. anatipestifer* ATCC11845 | CGCCGGGTACCATTCGGTCATTCAAACTTTATAGATACAGG |
| ExbB1P1 | *R. anatipestifer* ATCC11845 | AGGAGGAATTCACCATGGTATCTCAAACCACAAATACAG |
| ExbB2P1 | *R. anatipestifer* ATCC11845 | GGCGAGCTAGCAGGAGGTATGAATGTTTCAAACACGG |
| OMhemRP1 | *R. anatipestifer* ATCC11845 | TTTTGGGCTAGCAGGAGGAATTCACCATGATGCTGTTTTTCAGCACCGTTCTAAATG |
| OMhemRP2 | *R. anatipestifer* ATCC11845 | ATCCCCGAAGCTTATGGTTTAAAAATTAAATTGACAAG |
| TonB1P2 his | *R. anatipestifer* ATCC11845 | ATCCCCGAAGCTTATGGTTTAGTGGTGGTGGTGGTGGTGGAAAGTGATTTTGTAAGTGCC |
| TonB2P2 his | *R. anatipestifer* ATCC11845 | CCCAAGCTTGGGTTAGTGGTGGTGGTGGTGGTGATACTCAAAATTCATTGCC |
| TonB3P2 his | *R. anatipestifer* ATCC11845 | CGCCGGGTACCATTCGGTCAGTGGTGGTGGTGGTGGTGTTCAAACTTTATAGATACAGG |
| ErmRP1 | *R. anatipestifer* CH-1 | ACCACTTTCCAGTCTTACGAAG |
| ErmRP2 | *R. anatipestifer* CH-1 | CGACTTTGAACTACGAAGGATG |
| SpcRP1 | pAM238 | CTAGCTAGCTAGCTCGACTTCGCTGCTGCCC |
| SpcRP2 | pAM238 | CGGAATTCCGCGAATTGTTAGACATTATTTG |
| TonB1upP1 | *R. anatipestifer* ATCC11845 | CGGGATCCCGAGAAAGGGCTTAGCAGAATAG |
| TonB1upP2 | *R. anatipestifer* ATCC11845 | CTTCGTAAGACTGGAAAGTGGTAAGTTTACTTTTCTTGTACGG |
| TonB1downP1 | *R. anatipestifer* ATCC11845 | CATCCTTCGTAGTTCAAAGTCGAGCCAAATCAATAAAAGGATTTTAG |
| TonB1downP2 | *R. anatipestifer* ATCC11845 | GGGGTACCCCGTTTAAGTCATTTAGCCTTCTAGC |
| TonB2upP1 | *R. anatipestifer* ATCC11845 | CGGGATCCCGCGTTTAAGAGTCCTACAGG |
| TonB2upP2 | *R. anatipestifer* ATCC11845 | CTAGCTAGCTAGATAAAATATGAATTTTAAGGG |
| TonB2downP1 | *R. anatipestifer* ATCC11845 | CGGAATTCCGAATTCTGTTAAAATTATTATAAG |
| TonB2downP2 | *R. anatipestifer* ATCC11845 | GGGGTACCCCCACCTATATTATTCACAATATTTTC |
| 16SrRNAP1 | *R. anatipestifer* ATCC11845 | ATGCGAAAGGAGGATTGC |
| 16SrRNAP2 | *R. anatipestifer* ATCC11845 | TTACACCTCAAATACCTC |
| hi0933P1 | *R. anatipestifer* ATCC11845 | CGGCAGCTAATATAGACACC |
| hi0933P2 | *R. anatipestifer* ATCC11845 | AAGCGTGGGTTACATTACAG |
| ABCP1 | *R. anatipestifer* ATCC11845 | AGATACAGGTGGTAGCAGAC |
| ABCP2 | *R. anatipestifer* ATCC11845 | ATTACTCTAGCTCTGCCCTC |
